# Needle-free injection device for administration of cloprostenol to induce luteolysis in lactating dairy cows

**DOI:** 10.3168/jdsc.2025-0820

**Published:** 2025-09-04

**Authors:** Alyssa Leslie, Victor E. Gomez-Leon, Michael D. Kleinhenz, Mikaela Weeder, Ian Batey, Kennedy Kats, Bailey Fritz, Misty Bear, Scott Nordstrom, Serena Schotanus, Andrew Curtis, Santiago Paez Hurtado, Andreia Ferreira, Johann Coetzee

**Affiliations:** 1 Department of Clinical Sciences, College of Veterinary Medicine, Kansas State University, Manhattan, KS 66506; 2 Department of Animal Sciences and Industry, College of Agriculture, Kansas State University, Manhattan, KS, 66506; 3 Veterinary Education, Research and Outreach (VERO), Texas A&M University, Canyon, TX, 79015; 4 Department of Anatomy and Physiology, College of Veterinary Medicine, Kansas State University, Manhattan, KS 66506; 5 Merck Animal Health, Rahway, NJ 07065

## Abstract

The objective of this study was to evaluate the effectiveness of using a needle-free injection system for the intramuscular delivery of cloprostenol in lactating dairy cows. Twenty-six lactating Holstein dairy cows were randomly assigned to 1 of 3 treatments: (1) a single needle-free injection of 2 mL (0.5 mg) cloprostenol i.m. (NFI-PG; n = 10); (2) a single conventional needle injection of 2 mL (0.5 mg) cloprostenol i.m. (NDL-PG; n = 10); or (3) a needle-free injection of 2 mL physiological saline i.m. (CNTL, n = 6). Outcome measurements included corpus luteum (CL) size and blood flow via ovarian ultrasound, as well as progesterone (P4) and plasma cortisol (CORT) concentrations, all assessed at predetermined time points. Data were analyzed using a mixed model with the cow as the experimental unit, and fixed effects of group, day, and the group-by-day interaction. No significant differences were observed in P4 and CL outcome measures between the NFI-PG and NDL-PG groups. Both NFI-PG and NDL-PG cows exhibited similar decreases in P4 across all time points. In contrast, no significant changes in P4 concentrations and CL measures were observed in the CNTL group. Thus, the CNTL cows exhibited significantly higher P4 concentrations from 8 to 92 h compared with the NFI-PG and NDL-PG groups. Regression of CL, as indicated by a decrease in CL volume, blood flow, and P4 concentrations, was observed following both NDL-PG and NFI-PG injections, but not in the CNTL group. The NFI-PG cows had significantly lower CORT levels throughout the entire time period compared with both CNTL and NDL-PG cows. In conclusion, the use of a needle-free injection system for cloprostenol delivery resulted in similar outcomes to conventional needle injections, with comparable decreases in P4 and CL measures in lactating dairy cows and lower CORT concentrations.

## Highlights

• The aim was to test a needle-free injection system (NFI) to deliver cloprostenol in cattle.
• The needle-free injection system uses pressure to propel pharmaceuticals across various tissues and into the muscle.
• Progesterone, corpus luteum size, and blood flow decreased after cloprostenol delivery.
• NFI and traditional needle treatments were not different in effectively inducing luteolysis.

## Graphical Abstract

**Figure:**
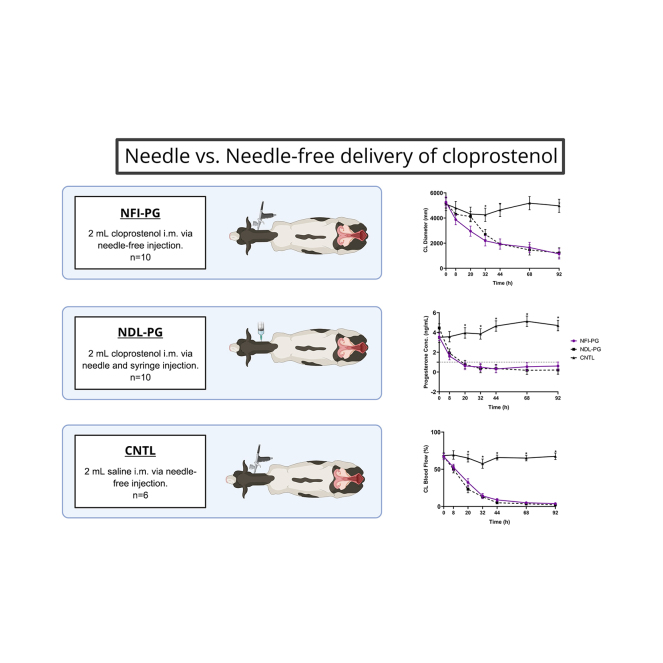
**Summary:** Needle-free system delivery of cloprostenol in cattle was validated by luteolysis based on corpus luteum (CL) volume, progesterone concentrations, and CL blood flow. i.m. = intramuscularly.

Estrous synchronization is a tool that allows dairy operations to control the estrous cycle of the cow and facilitate timed insemination. The simplest synchronization protocols rely only on treatment with PGF_2α_ or its analogs to regress the corpus luteum and allow spontaneous estrus to occur with ovulation to follow. More comprehensive programs such as Double-Ovsynch and PG3-G + Ovsynch require the combination of up to 6 or 7 administrations of PGF_2α_ and GnRH analogs. The administration of these reproductive hormones may be performed via i.m., s.c. (Chebel et al., 2007), i.v., intravaginal, or intravulvar administration (Masello et al., 2020a,b). Still, i.m. or s.c. administration is preferred due to its ease of administration, effectiveness, and regulatory approval as the route of administration (Colazo et al., 2002). Conventional methods of i.m. administration are done via needle and syringe. However, needle-free injection (**NFI**) might be more advantageous for employee safety and decreased waste generation. Various versions of NFI systems have been created (Curtis et al., 2024). Conventional NFI methods use mechanical force to administer the drug to a patient via a compressed spring or compressed gas. Using a piston with conventional NFI methods enable the drug to be pushed through a nozzle at a high velocity, ultimately traversing the different layers of tissue based on the amount of force behind the piston (Schoppink and Fernandez Rivas, 2022). Previously, NFI for i.m. administration have been used in humans (Giudice and Campbell, 2006) and swine (Babiuk et al., 2003). In humans, NFI allows for impressive efficiency for vaccinators and alleviates trypanophobia and has also been noted to be less painful than a conventional needle and syringe (Igarashi, 2006). In swine, NFI is commonly used to administer vaccinations in large operations. Additionally, the risk of broken needles occurring in the animal is eliminated, allowing for a safer and higher quality meat product at slaughter (Chase et al., 2008). Furthermore, NFI has been shown to aid in mitigating the transfer of blood-borne pathogens such as *Anaplasma marginale* in cattle (Reinbold et al., 2010).

Currently, minimal data are available to support the use of NFI in the administration of prostaglandin analogs in lactating dairy cattle. Thus, herein we propose a pilot study to test the efficacy of luteal regression in response to i.m. cloprostenol administered using NFI. We hypothesize that NFI of cloprostenol will induce luteal regression in lactating dairy cows. The Institutional Animal Care and Use Committee at Kansas State University approved this study (protocol #4722).

A total of 26 lactating Holstein dairy cows with synchronized estrous cycles were used. The cattle were housed Kansas State Dairy Research Center (Manhattan, KS) in outdoor, covered freestall sheds bedded with sand and were fed a TMR twice daily. The TMR was formulated to meet or exceed the nutrient requirements for lactating dairy cows producing 50 kg of milk per day with 3.5% milk fat (NASEM, 2021). Cows were milked 3 times per day. Before study enrollment, all animals were evaluated for normal body temperature and normal ovarian function. Cows were enrolled at 49 ± 3 DIM and received a controlled internal drug release insert (**CIDR**; Eazi-breed CIDR, Zoetis Animal Health, Parsippany, NJ) containing 1.38 g of progesterone, along with an i.m. injection of 172 μg of gonadorelin (Fertagyl, Merck Animal Health, Rahway, NJ). The CIDR were removed between 56 ± 3 DIM, and cows were administered an i.m. injection of 500 µg of cloprostenol (Estrumate, Merck Animal Health, Rahway, NJ). Approximately 8 ± 3 h later, the cows received a second i.m. injection of 500 µg of cloprostenol to ensure luteolysis. Finally, at 58 ± 3 DIM, cows were administered an i.m. injection of 86 µg of gonadorelin, completing the estrous synchronization protocol.

Eight days after the gonadorelin administration (at 66 ± 3 DIM), ultrasound was used to confirm the presence of an active corpus luteum (**CL**), defined as ≥17 mm in diameter (Colazo et al., 2002; Répási et al., 2003; Cuervo-Arango et al., 2011) with >25% blood flow (Andrade et al., 2024), before randomization into the treatment groups. On this day (66 ± 3 DIM), a random number generator (random.org) was used to assign each cow to 1 of 3 treatments: needle-free system injection of cloprostenol (**NFI-PG**; n = 10), conventional needle injection of cloprostenol (**NDL-PG**; n = 10), or needle-free injection of physiological saline (**CNTL**; n = 6), administered at the start of the sample collection period. Cows assigned to the NFI-PG group received 500 µg (2 mL) of cloprostenol using the **NFI** (Pulse 250, Pulse Needlefree Systems, Lenexa, KS). The NFI was set at 552 to 621 kPa per the manufacturer's recommendations, delivering the cloprostenol intramuscularly. Cows assigned to the NDL-PG group received 500 µg (2 mL) of cloprostenol using a 3-mL syringe and 18-gauge × 3.81 cm (1 1/2 in.) needle. Once the needle and syringe were placed intramuscularly, the syringe was aspirated to ensure correct placement and lack of blood. Upon confirmation, the cloprostenol was administered. Cows assigned to the CNTL group received 2 mL of sterile saline (0.9% Sterile Saline, VetOne, Boise, ID) using the NFI. The same NFI parameters as the NFI-PG group were used for the CNTL group. All injections were administered on the right side of the cow's neck following Beef Quality Assurance guidelines (BQA, 2019). During the dosing period, cows were restrained in a box stall with a headlock gate to ensure the safety of both the personnel and the animal. Only personnel administering treatments were unblinded to treatment allocation. All other study personnel assisting with data collection and analysis were blinded to treatment allocation.

Data collection parameters included collecting blood samples to assay cortisol and progesterone concentrations, CL diameter, and CL blood flow. Collection for cortisol occurred at 0, 2, 4, 6, 8, 10, 12, 15, 20, 30, 45, 60, 90, and 120 min. Blood samples for progesterone analysis and ovarian ultrasonography occurred at 0, 8, 24, 32, 44, 68, and 92 h. For this, cattle were placed in a box stall and restrained 30 min using a headlock and a halter 30 min prior to collection. Catheters were placed 15 min before collection in the right jugular vein of the cattle to aid in the intensive sample collection and minimize stress. The catheter site was first prepared by injecting 1 mL of 2% lidocaine (VetOne, Boise, ID) at the intended site of catheter insertion. Following the lidocaine, the site was clipped and aseptically scrubbed using chlorhexidine surgical scrub (Chlorhexidine 4%, VetOne, Boise, ID) and cotton gauze soaked in 70% isopropyl alcohol (Vedco, St. Joseph, MO). A 14-gauge by 13-cm catheter was used for each cow (MILACATH, MILA International Inc., Florence, KY). The catheter was placed into the jugular vein and a cap (FiveTen-K Male Adapter Plug, VetOne, Boise, ID) was placed on the catheter. Catheters were sutured to the skin using 0 nylon suture (Securolon, Securos Surgical, Fiskdale, MA). Catheters were flushed with 2 mL of 0.9% sterile saline (VetOne, Boise, ID) at 0, 2, 4, 6, 8, 10, 12, 15, 20, 30, 45 min. Heparinized saline (Heparin Lock Flush Syringe 10 units/mL, Medefil Inc., Glendale Heights, IL) was administered in between time points 60, 90, and 120 min. A total of 12 mL of blood was collected from the catheters at each time point. Catheters were removed following the sample collection at 120 min, and cows were then returned to their respective group pens. After blood collection, blood tubes were placed on ice and transported to the laboratory, where blood samples were centrifuged at 1,500 × *g* for 10 min at 4°C. The plasma was pipetted off from the top of the tube, taking precautions not to disrupt the buffy coat. The plasma was then transferred into prelabeled cryotubes and placed in a −80°C freezer to be stored until analyzed.

Plasma cortisol concentrations were measured using methods from Martin et al. (2022). Samples were analyzed in duplicate utilizing a commercially available RIA antibody-coated tube kit (MP Biomedicals, Irvine, CA). Blank 12 × 75 mm polypropylene tubes were used to measure nonspecific binding. The RIA was completed following manufacturer specifications with minor modifications as described by Martin et al. (2022); the standard curve was extended to include 1 and 3 ng/mL. The standard curve ranged from 1 to 300 ng/mL. A low (25 ng/mL) and high (150 ng/mL) quality control (**QC**) were run at the beginning and end of each set to determine interassay variability. Volume for standards, QC, and samples was adjusted to 50 µL. Sample incubation occurred at room temperature for 30 min before the addition of ^125^I. After sample incubation, the ^125^I tracer was added to each tube and the manufacturer protocol was followed for the rest of the assay. Samples were placed on a gamma counter (Wizard2, PerkinElmer, Waltham, MA) and counted approximating 1 min per sample. Raw data from the gamma counter was uploaded into the MyAssays Desktop software (version 7.0.211.1238, MyAssays, Brighton, UK). Cortisol concentrations were determined with the MyAssasys Desktop software and standard curves were plotted using a 4-parameter logistic curve. Interassay and intra-assay variation were determined to be 19.74% and 10.17%, respectively.

For blood collection of P4 determination, cattle were moved into a 4-cow palpation rail, and blood samples were harvested using coccygeal vein venipuncture. Samples were centrifuged at 1,500 × *g* for 10 min at 4°C. The plasma was pipetted off from the top of the tube, taking precautions not to disrupt the buffy coat. The plasma was then transferred into prelabeled cryotubes and placed in a −80°C freezer to be stored until analyzed.

Plasma progesterone concentrations were determined using a commercially available RIA antibody-coated tube kit (MP Biomedicals, Irvine, CA) following manufacturer specifications with minor modifications. The standard curve ranged from 0.015 to 80 ng/mL. A low (0.25 ng/mL) and high (40 ng/mL) QC were run at the beginning and end of each set to determine interassay variability. Volume for standards, QC, and samples was adjusted to 50 µL. Sample incubation occurred at room temperature for 30 min before the addition of ^125^I. After the incubation period, ^125^I was added to each tube, and the manufacturer protocol was used for the remainder of the assay. Samples were placed on the gamma counter and counted as described for the cortisol assay. Progesterone concentrations were determined with the MyAssays Desktop software and standard curves were plotted using a 4-parameter logistic curve. The interassay and intra-assay variation were determined to be 10.21% and 15.70%, respectively.

Ultrasonography of the ovaries was conducted to determine accurate CL volume and blood flow at 0, 8, 20, 32, 44, 68, and 92 h relative to PGF treatments. Ultrasonography was conducted with an Ibex EVO II (E.I. Medical Imaging, Loveland, CO). Diameter measurement was taken at the largest apparent CL image using the caliper tool of the machine. Two perpendicular diameters of each CL were documented and then averaged. Blood flow was measured using the ultrasound Doppler mode. Cows with multiple CL were included in the study, and the means of the CL blood flow and volumes were used for statistical analysis. The CL volume was calculated using the sphere volume formula: V = 4/3 × π × R^3^, where R = (CL length/2 + CL width/2)/2, as previously described (Sartori et al., 2002). If present, the CL cavity volume was calculated and subtracted from the CL volume. For cows with 2 CL, the CL volume represents the volume of CL1 + the volume of CL2.

Plasma cortisol and progesterone concentrations were log-transformed for normality before statistical analysis. Analysis was conducted using statistical software (JMP Pro 17.0, SAS Institute Inc., Cary, NC). Outcome measures included plasma cortisol concentrations, plasma progesterone concentrations, CL volume, and CL blood flow. Data were analyzed using a mixed model with the individual animal as the experimental unit. Random effect was the treatment, and time, treatment-by-time interaction, and enrollment date were fixed effects. Significance was set at a *P* ≤ 0.05 a priori*.* All outcomes are reported as LSM ± SEM.

Results for outcomes are presented in Table 1. No difference was observed in CORT concentrations (ng/mL) at 0 h, before treatment administration. The NFI-PG group exhibited significantly lower CORT compared with both the NDL-PG and CNTL groups (*P* < 0.0001). This observed treatment effect was significant (*P* = 0.04). A time effect was observed in which CORT concentrations increased immediately after treatment administration and decreased from the 60 min to 120 min time points, regardless of the group. Cortisol concentrations fluctuated throughout the study, but the NFI-PG group consistently maintained significantly lower cortisol concentrations over the 4, 6, 12, 20, 30, 45, 60, 90, and 120 min time points (*P* < 0.0001). Despite these measurements, no significant treatment-by-time effect was observed (*P* < 0.46).

**Table 1:** Overall treatment LSM ± SEM for outcome measures for cows receiving cloprostenol via the needle-free injection device (NFI-PG), cloprostenol via conventional needle and syringe (NDL-PG), or saline via needle-free injection device (CNTL)

Variable	CNTL, n = 6	NFI-PG, n = 10	NDL-PG, n = 10	*P-*value		
				Treatment	Time	Treatment × time
Cortisol (ng/mL)	26.65 ± 4.78^a^	14.43 ± 3.53^b^	24.95 ± 3.36^a^	0.04	<0.0001	0.46
Progesterone concentration (ng/mL)	4.16 ± 0.42^a^	0.78 ± 0.29^b^	0.61 ± 0.33^b^	0.0007	<0.0001	<0.0001
CL volume (mm^3^)	4,752.50 ± 427.87^a^	2,715.53 ± 330.95^b^	2,987.60 ± 330.95^b^	0.0026	<0.0001	<0.0001
CL blood flow (%)	62.26 ± 5.65^a^	24.89 ± 4.38^b^	27.00 ± 4.38^b^	<0.0001	<0.0001	<0.0001

a,b Within rows, different superscripts indicate a significant treatment difference at *P* < 0.05.

No difference was observed in P4 concentrations (*P* = 0.88) at 0 h, before treatment administration among NFI-PG (3.55 ± 0.38 ng/mL), NDL-PG (4.46 ± 0.41 ng/mL), and CNTL (3.51 ± 0.53 ng/mL). Moreover, all cows in the study had P4 concentrations above 1 ng/mL at 0 h (Santos et al., 2010). Following cloprostenol administration, 2 cows in the NFI-PG group and 1 cow in the NDL-PG group failed to fall below 1 ng/mL of P4, indicating failure to completely regress their CL. None of the cows in the CNTL group had a decrease in P4 concentrations or fell below 1 ng/mL. In general, there were no differences among the NFI-PG and the NDL-PG treatment groups, between 0 h to 92 h for progesterone concentrations (*P* = 0.52), as seen in Figure 1A. The observed treatment effect between the CNTL group and the 2 cloprostenol groups was significant (*P* < 0.0001). The interaction of group by time was significant, mainly due to similar P4 concentration among the groups at 0 and 8 h and lower concentrations in the NFI-PG and NDL-PG from 20 to 92 h when compared with the CNTL group.

**Figure 1:**
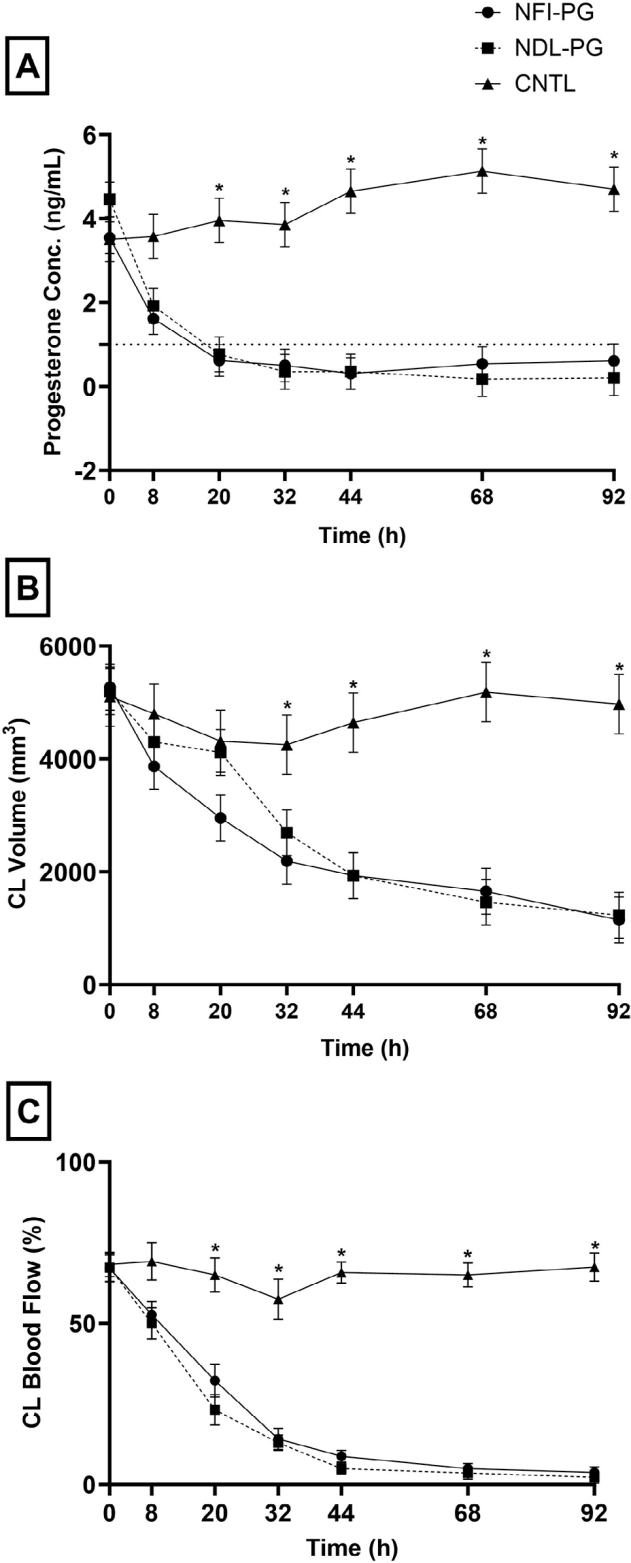
Mean progesterone concentrations (Conc.; ng/mL, panel A), mean CL volume (mm^3^, panel B), and mean CL blood flow (%; panel C) over time for cows receiving cloprostenol via the needle-free injection device (NFI-PG; n = 10), cloprostenol via conventional needle and syringe (NDL-PG; n = 6), or saline via needle-free injection device (CNTL; n = 6). Error bars indicate SEM. *Denotes differences within time points (*P* ≤ 0.05).

No differences were observed in CL volume (mm^3^) at 0 h, before treatment administration. No differences were detected in CL volume between the NFI-PG and NDL-PG groups from 0 h to 92 h (*P* = 0.57*)*. At 20 h, the CL volume of the NFI-PG group (2,954.02 ± 407.91 mm^3^) tended to be smaller than the NDL-PG (4,112.17 ± 407.91 mm^3^) and CNTL groups (44,313.97 ± 550.29 mm^3^; *P* = 0.07; Figure 1B). Although both treatment methods were effective in inducing a decrease in CL volume, the absence of treatment in the CNTL group resulted in no CL volume change, as expected (*P* = 0.32). The analysis denoted a significant time effect, observing that as time increased, CL volume decreased (*P* < 0.0001). A treatment-by-time interaction was also observed, with CL volumes of NFI-PG and NDL-PG being lower than the CNTL starting at 32 h and continuing through 92 h (*P* < 0.0001).

We found no differences in CL blood flow (%) at 0 h, before treatment administration. No difference was observed between the NFI-PG and NDL-PG groups (*P* = 0.94), but the CNTL group experienced a smaller change in blood flow between 0 h and 92 h, which was significant (*P* < 0.0001; Figure 1C). The effect of treatment on CL blood flow was observed to be significant (*P* < 0.0001). A time effect was observed for blood flow, indicating that as time increased, the change in blood flow decreased, with all time points being lower than baseline (*P* < 0.0001). A treatment-by-time point interaction was observed with the NFI-PG and NDL-PG groups having lower CL blood flow compared with CNTL cows starting at 20 h and continuing through 92 h time point (*P* < 0.0001).

In this study, we compared the use of an NFI system and a conventional needle and syringe method for administering cloprostenol to lactating dairy cows. Reproductive management plays a substantial part in the economic potential of a dairy herd (Consentini et al., 2021). Producers can maximize the economic potential within their operations by using reproductive management drugs such as cloprostenol. Based on the results of the current study, the NFI system would allow producers to administer cloprostenol intramuscularly as effectively as a needle and syringe. The effectiveness of the NFI system in delivering cloprostenol in lactating dairy cows was demonstrated by the observed decreases in progesterone concentrations, CL volume, and CL blood flow.

At enrollment, all cows in this study had CL present and progesterone concentrations above 1.0 ng/mL, indicating the CL was mature and active. A response to cloprostenol treatment threshold of 1 ng/mL was selected based on previous work by (Santos et al., 2010). Two of the NFI-PG cows and one NDL-PG cow failed to respond to cloprostenol based on their progesterone concentrations. These cows all had decreases in progesterone concentrations and CL blood flow following cloprostenol administration. The need for 2 doses of cloprostenol to completely regress CL and achieve P4 below 1 ng/mL and higher fertility has been illustrated (Wiltbank et al., 2015). However, using 2 cloprostenol injections would have potentially biased the physiologic parameters assessed in our study. Instead, we extended one extra day of the protocol: 8 instead of 7 d between gonadorelin and cloprostenol.

Several studies have tested administering PGF and its analogs at different doses, frequencies, intervals, and through different routes to simplify management or maximize luteolysis (Colazo et al., 2002; Wiltbank et al., 2015). Nonetheless, most tested approaches consist of using a needle to administer the drugs. Reported needle-less approaches in the literature include intravaginal instillation by using a uterine infusion catheter or an automated controlled-release device (Masello et al., 2020a,b). Both of these systems have been effective in inducing luteolysis (P4 <1 ng/mL) at 48 h after treatment. Similarly, in our study, the NFI-PG group achieved P4 concentrations <1 ng/mL at a similar time (Figure 1A). This was also evidenced by the diminishment of CL volume and CL blood flow in the NFI-PG.

One of the primary motives for exploring NFI systems in this context was to enhance occupational safety. Needle-stick injuries pose a substantial risk to employees involved in veterinary care, including dairy farm workers. The NFI system has a built-in safety mechanism to prevent accidental injection, requiring the user to manually unlock the triggering device before applying the pressure needed to dispense the medication. Using NFI systems may also benefit animal health, as previous studies have suggested the potential for reducing the transmission of blood-borne pathogens (Reinbold et al., 2010). Although this study did not specifically measure pathogen transmission, adopting NFI systems could enhance overall herd health management by reducing the spread of pathogens such as *Anaplasma marginale* and bovine leukosis virus. Additionally, the NFI method could economically benefit large-scale operations by lowering the recurring costs of needles and syringes. In addition to animal health and economic advantages, animal welfare is another benefit of the NFI. This study highlighted lower cortisol levels in cows receiving cloprostenol via the NFI compared with conventional needle and syringe methods, which could indicate reduced stress with the NFI. Further research is needed to establish a definitive correlation between low stress and NFI usage for cloprostenol injection.

In conclusion, this study sought to validate a needle-free device to induce luteolysis by delivering cloprostenol i.m. in lactating dairy cows. The NFI was compared with the conventional needle injection and a saline administration using the NFI. Regression of CL, as indicated by a decrease in CL volume, blood flow, and P4 concentrations, was observed following both cloprostenol administration with the NFI and needle injection, but not in the group receiving saline. In fact, mean plasma progesterone concentrations and CL measurements were not different in cattle receiving NFI of cloprostenol when compared with the conventional needle and syringe administration of cloprostenol in lactating dairy cows. Additionally, NFI of cloprostenol offered lower plasma cortisol concentrations than needle and syringe administration of cloprostenol in lactating dairy cows. Continued research and practical adoption efforts are warranted to fully realize the potential of NFI technologies in veterinary medicine and animal production systems.

## References

[bib1] Andrade J.P.N., Domingues R.R., Monteiro P.L.J., Dias J.R., Pimenta C., Guimarães A.S., Barbosa L., Merhi S., Sartori R., Wiltbank M.C. (2024). Identification of nonpregnant beef cows based on CL size vs. luteal blood perfusion at 21 days after timed artificial insemination. Theriogenology.

[bib2] Babiuk S., Baca-Estrada M.E., Foldvari M., Baizer L., Stout R., Storms M., Rabussay D., Widera G., Babiuk L. (2003). Needle-free topical electroporation improves gene expression from plasmids administered in porcine skin. Mol. Ther..

[bib3] BQA (Beef Quality Assurance) (2019). National Manual. https://www.bqa.org/Media/BQA/Docs/nationalmanual.pdf.

[bib4] Chase C.C.L., Daniels C.S., Garcia R., Milward F., Nation T. (2008). Needle-free injection technology in swine: Progress toward vaccine efficacy and pork quality. J. Swine Health Prod..

[bib5] Chebel R.C., Santos J.E., Rutigliano H.M., Cerri R.L. (2007). Efficacy of an injection of dinoprost tromethamine when given subcutaneously on luteal regression in lactating Holstein cows. Theriogenology.

[bib6] Colazo M.G., Martinez M.F., Kastelic J.P., Mapletoft R.J. (2002). Effects of dose and route of administration of cloprostenol on luteolysis, estrus, and ovulation in beef heifers. Anim. Reprod. Sci..

[bib7] Consentini C.E.C., Wiltbank M.C., Sartori R. (2021). Factors that optimize reproductive efficiency in dairy herds with an emphasis on timed artificial insemination programs. Animals (Basel).

[bib8] Cuervo-Arango J., García-Roselló E., García-Muñoz A., Valldecabres-Torres X., Martínez-Ros P., González-Bulnes A. (2011). The effect of a single high dose of PGF2α administered to dairy cattle 3.5 days after ovulation on luteal function, morphology, and follicular dynamics. Theriogenology.

[bib9] Curtis A.K., Weeder M.M., Martin M.S., Leslie A.A., Montgomery S.R., Johnson B.T., Coetzee J.F., Lou M.E., Viscardi A.V., Kleinhenz M.D. (2024). A novel needle-free method of lidocaine administration during standing castration of Holstein bulls. JDS Commun..

[bib10] Giudice E.L., Campbell J.D. (2006). Needle-free vaccine delivery. Adv. Drug Deliv. Rev..

[bib11] Igarashi Y. (2006). Clinical evaluation of the needle-free injection system VISION® for growth hormone therapy in children. Clin. Pediatr. Endocrinol..

[bib12] Martin M.S., Kleinhenz M.D., Edwards-Callaway L.N., Engle T.E., Guimaraes O., Schafer D.W., Montgomery S.R., Curtis A.K., Weeder M.M., Jacobs D.R., Coetzee J.F. (2022). The effect of breed, sex, and oral meloxicam administration on pain biomarkers following hot-iron branding in Hereford and Angus calves. J. Anim. Sci..

[bib13] Masello M., Ren Y., Erickson D., Giordano J.O. (2020). An automated controlled-release device for intravaginal hormone delivery. JDS Commun..

[bib14] Masello M., Scarbolo M., Schneck M., Perez M., Schillkowsky E., Sitko E., Hernandez F.S., Cabrera E., Rossi R., Giordano J. (2020). Intravaginal instillation of prostaglandin F_2α_ was as effective as intramuscular injection for induction of luteal regression in lactating dairy cows. J. Dairy Sci..

[bib15] NASEM (National Academies of Sciences, Engineering, and Medicine) (2021). https://doi.org/10.17226/25806.

[bib16] Reinbold J.B., Coetzee J.F., Hollis L.C., Nickell J.S., Riegel C.M., Christopher J.A., Ganta R.R. (2010). Comparison of iatrogenic transmission of *Anaplasma marginale* in Holstein steers via needle and needle-free injection techniques. Am. J. Vet. Res..

[bib17] Répási A., Beckers J.F., Sulon J., Perényi Z., Reiczigel J., Szenci O. (2003). Effect of different doses of prostaglandin on the area of corpus luteum, the largest follicle and progesterone concentration in the dairy cow. Reprod. Domest. Anim..

[bib18] Santos J.E.P., Narciso C.D., Rivera F., Thatcher W.W., Chebel R.C. (2010). Effect of reducing the period of follicle dominance in a timed artificial insemination protocol on reproduction of dairy cows. J. Dairy Sci..

[bib19] Sartori R., Rosa G.J.M., Wiltbank M.C. (2002). Ovarian structures and circulating steroids in heifers and lactating cows in summer and lactating and dry cows in winter. J. Dairy Sci..

[bib20] Schoppink J., Fernandez Rivas D. (2022). Jet injectors: Perspectives for small volume delivery with lasers. Adv. Drug Deliv. Rev..

[bib21] Wiltbank M.C., Baez G.M., Cochrane F., Barletta R.V., Trayford C.R., Joseph R.T. (2015). Effect of a second treatment with prostaglandin F_2α_ during the Ovsynch protocol on luteolysis and pregnancy in dairy cows. J. Dairy Sci..

